# Retraction: Upregulated circular RNA VANGL1 contributes to progression of non-small cell lung cancer through inhibition of miR-195 and activation of Bcl-2

**DOI:** 10.1042/BSR-2018-2433_RET

**Published:** 2022-12-22

**Keywords:** apoptosis, circular RNA VANGL1, non-small cell lung cancer

This article is being retracted from *Bioscience Reports* at the request of the authors due to concerns over the reliability of the results. The Editorial Office and the authors were alerted by a reader to apparent similarities between images in this manuscript with those from other manuscripts (from unrelated authors), which include the following:

- Figure 5C GAPDH/A549 bands and the GAPDH bands of both Fig. 5F from Liu et al. 2020 (doi: 10.1042/BSR20191330) and Fig. 4L from Fu et al. 2019 (doi: 10.18632/aging.102325),
- The Figure 3 A549 migration/si-NC, H1299 migration/si-circVANGL1 and the H1299 invasion/si-circVANGL1 panels with those from Figs. 6D and 7F from Yang et al. 2020 (doi.org/10.3389/fonc.2020.01104),
- Figure 2A (A549 and H1299/Bcl-2 and GAPDH bands) with bands from Fig. 3D from Sun et al. 2017 (doi: 10.18632/oncotarget.15846),
- The Figure 6A A549/GAPDH band with bands in both Fig. 3F from Chen et al. 2019 (doi: 10.1042/BSR20191050) and Fig. 4C from Li et al. 2019 (doi: 10.1042/BSR20181882).

The authors have been unable to find the original data and are therefore unable to correct the article. They wish to retract the article. The Editor-in-Chief and Editorial Board agree with the retraction.

